# A New Departure

**Published:** 1871

**Authors:** 


### Editorial and Miscellaneous.

### *A New Departure.*

Whatever may be the conflicting views in regard to the merits of the political "New Departure" which has excited so much discussion, there can be no difference of opinion among those who feel an interest in the advancement of medical science and the relief of suffering humanity as to the present and prospective improved status of the medical profession in Georgia, in their determination to waste no more time in conflicts which bring them into discredit with their brethren in other States and greatly lower the respect and confidence of the people.

It is with great pleasure we record this *new departure* from controversy and strife to the peaceful walks of science, and to the pursuit of the high objects which we profess to have in view.

For ourselves, with the honest purpose of discharging the claims of right and justice, and in the interests of peace and harmony, and with the view of repairing any injury which may have been inflicted by our hands in the course of an exciting controversy, and of securing permanent fraternal relations among those who should be joined hand in hand in the advancement of a noble calling, everything which could be construed into offensive reflections upon the organized profession of the State has been *fully, freely and honestly retracted.* This action upon our part having been accepted, and a settlement of all professional difficulties in which we have been involved, having been made, we have no farther controversy with any medical man, but extend the right hand of fellowship to all.

That there may be a few dissatisfied individuals is doubtless true, and that we have personal enemies who would not be satisfied with *any compromise* we regret to know, but these facts do not in any way interfere with the demonstrated fact that the

*medical profession of Georgia are now at peace*, and are determined no longer to be a reproach to the cause of science and humanity.

In our city we can already see the most marked evidences of the good results following the settlement of these troubles. Gentlemen are beginning to understand each other and to recognize the fact that it is better to have fraternal relations rather than enmity, even though some sacrifices have to be made upon both sides to obtain the desired result, and that in all honorable compromises each party to a contest must yield something. Time that was formerly spent in warring upon each other is now being devoted to general and individual advancement in the profession and to the promotion of good will.

With the no distant future will be still more strongly developed the fact that we have no views or interests which are antagonistic to the highest requisitions of the medical profession of this State or of the United States, determined as we are, so far as we can have any agency in the matter, to place medical teaching and medical literature upon the highest possible elevation that can be demanded, and to uphold no narrow or selfish policy that looks alone to the advancement of our own or any other individual interest.

Upon this basis we cordially invite every medical man who is honestly and earnestly devoted to the best interests of his calling to unite with us and to accompany us in this "new departure," and to aid in building up a system of medical education and a medical literature that will be an honor to the State and to the country.

We have reason to know that it is the determination of the members of the Georgia Medical Association to make the next meeting of that body *memorable* as the commencement of a new era in its history by large and valuable contributions to science, confining itself rigidly to its proper functions as a scientific body, eschewing all attempts at judicial regulations which will be left to some other organization for which provision may be made as proposed in an amendment offered at the last meeting, or to one of a more general character which has been proposed as a *Court* to relieve the American Medical Association of attempts at judicial regulations which has, as admitted by all, very nearly resulted in its entire ruin.

We are sure that the future meetings of the Association will be productive of much good, and that the large mass of the profession who have declined to attend its annual gatherings, since the war, will no longer be deterred from active participation in its deliberations, from the apprehension of being forced into some bitter, personal contest.

